# Hospital and Institutional News

**Published:** 1912-07-13

**Authors:** 


### HOSPITAL AND INSTITUTIONAL NEWS.

### THE KING IN DOCKLAND.

General regret is felt that the King has thought it fit, on the advice of his Ministers, to cancel his proposed visit to the East End of London. It is to be hoped that this cancellation is merely a postponement of the visit. Since his Majesty's visit to the London Hospital after his accession to the throne East London has had no opportunity of seeing the King and Queen in its own stronghold of rapidly altering slumdom. Whatever reasons may have induced the present cancellation, it is safe to state that there is a feeling that the visit should be made at a later period when the local conditions are less regrettable than they are at present. To an outsider wandering in the region of the wharfs and docks it seems that there is no interruption of work and that there is no strike. But there is undoubtedly a great deal of distress. Attempts are being made to cope with the poverty and starvation, but some of these philanthropic efforts are impeded and hampered by the strikers themselves. It is surely the first time in history when hungry women threw bread into the gutter because the provisions had been sent to them in vehicles whose drivers they did not like.

### THE LONDON SCHOOL OF TROPICAL MEDICINE AND THE KING'S VISIT.

The authorities of the London School of Tropical Medicine have received an official intimation of the fact that the King has cancelled the visit to the Royal Albert Dock Hospital. His Majesty was to have visited the school on the 17th inst. to lay the foundation-stone of the new block. In the circumstances the arrangements have been cancelled. There will be no official ceremony in connection with laying the foundation-stone, but we understand building operations will not be delayed in consequence. His Majesty's decision is obviously due to the same reasons that have led to the postponement of the visit to the East End. The strike still exists, and while it lasts there cannot very well be ceremonial functions inside the district patrolled by the pickets. The school and the hospital attached to it are so well known, both to his Majesty and to the general public, that it is more than likely that the cancellation of the visit will bring them tokens of practical sympathy from all sides. A meeting in support of the school will be held at the Foreign Office on July 17, the day on which the foundation-stone was to have been laid. Mr. Austen Chamberlain will preside, and the Colonial Secretary, Sir Edward Grey, and Lord Crewe will be among the speakers.

### THE ADVENT OF THE INSURANCE ACT.

On Monday next, the 15th inst., the Insurance Act comes into force. During the past two months hospital secretaries all over the country have been trying to understand what their duties are in connection with this measure. The Insurance Commissioners have presented them with special pamphlets and placards, but it cannot honestly be stated that these explanatory memoranda have really been of assistance to those who have only tried to master the Act during the past quarter. It may be doubted if there is anyone who really comprehends the mysterious clauses in all their detail. Of course, there are always the Commissioners to refer to. Anyone can write to them for information, and the letters need not even be stamped; it is sufficient to put "O.H.M.S." on the envelopes. When the public realises its potential in this matter of asking for information the Commissioners are likely to have a very busy time. There are so many points on which expert advice is necessary that no one can be blamed for going to headquarters direct. The composition and strength of the Commissioners will be severely tested in the course of the ensuing three months. With most hospital secretaries we think the Act clumsy and inchoate, but it is upon us at last, and the public has to make the best of the matter. That being the case, our own Insurance Act department, which has already answered a large crop of questions, is likely to be more in request than ever. Arrangements have been made to cope fully with all further demands upon it.

### "CHARITY SHOWS AND THEIR ABUSES."

Under this heading *The Gentlewoman* comments frankly and incisively upon the eternal mismanagement of charity shows which are ostensibly organised in aid of philanthropic institutions, but are in reality society functions. The main theme of the criticism is the recent ball held in aid of the funds of the Soldiers' and Sailors' Help Society—an undertaking which probably cost some tens of thousands, and yielded a couple of thousands to the charity in whose favour it was organised. It is true there is a certain advertising value in such shows which cannot definitely be calculated in pounds, shillings, and pence, but such advertising or publicity profit may be obtained much more cheaply and much more effectively by consultation with an up-to-date agent than by promoting balls or festival shows, the initial cost of which far exceeds any possible profit. Our contemporary makes some well deserved remarks on the paid organiser and helper who generally manages to secure something out of the benefit performance in which he or she participates. "Philanthropic efforts which usually meant in the old days self-sacrifice of time and money all round have produced a somewhat reprehensible type of worker—*i.e.*, the person who 'voluntarily' organises a charity in order to see how much he or she can make out of it. It is well known that this scandal has been going on for years past, and opponents of it have submitted quietly to the obvious 'ramp' in order that the public may not be estranged from a project. The 'charity showmongers' who start and organise these projects for personal profit have blossomed forth, however, in so many places that it is time an honest attempt was made to stamp them out." This is a conclusion with which every hospital worker will cordially agree.

### THE SUFFRAGIST HOSPITAL-SUPPORTER.

The excuse made by a lady subscriber that she could no longer support the Metropolitan Hospital "because of the heavy sacrifices she felt called upon to make for the cause of women's enfranchisement" will hardly, we hope, find imitation among her co-suffragists. The Hon. Reginald Ailwyn Fellowes, who presided at the anniversary dinner of the hospital on Monday night last, told the story, and mentioned that another subscriber had retired from the subscription list because of the recent increased taxation. Rightly, the Chairman refuses to accept either excuse as valid. If the suffragist supporters would devote half of the money, now spent in paying fines for the breaking of windows belonging to inoffensive private firms or individuals, to founding a woman's cot, or even a W.S.P.U. bed in a metropolitan or provincial hospital, they would advertise their cause permanently, and at the same time be assured that they are benefiting others. Indeed, it is remarkable that the women who do most in the militant agitation have done the least in support of the hospitals. The Metropolitan Hospital does excellent work in a district where it is needed. It is one of the few London hospitals that specially provide for the admission and convenience of Jewish patients, and those who still imagine that London requires a special Jewish hospital are recommended to visit the Metropolitan, and see for themselves in what way this institution deals with its Jewish patients. At Monday's dinner the Secretary, Mr. Buchanan, stated that the total amount received in subscriptions was £9,000. The hospital and its secretary are to be congratulated upon its progress and the efforts it has made during the last twelve months to improve its accommodation and increase its working powers.

### LONDON VACANCIES.

Another addition to the fairly long list of present vacancies on the honorary visiting staffs of the London hospitals is that of surgeon to out-patients at the Hampstead General and North-West London Hospital at Haverstock Hill. This vacancy is caused by the resignation of Mr. W. Stephen Fenwick, who was recently elected to the staff of Charing Cross Hospital. Mr. Fenwick is also senior assistant surgeon at the Queen's Hospital for Children at Hackney and assistant-surgeon to the Gordon Hospital for Rectal Diseases.

### WHAT WILL THE GOVERNMENT DO IN REGARD TO CONSUMPTION?

Although there is to be a fairly long latent period before the medical and sickness benefits under the Insurance Act come into force, there is no such statutory delay authorised in connection with the sanatorium benefit. This is, indeed, supposed to commence at once, but as far as can be seen at present the Government is not in the least able to carry out its promises in this direction. It is difficult to see how the Insurance Commissioners can avoid assuming, temporarily at least, the position of defaulters. The Government proposed to spend a capital sum of £1,600,000 in setting up sanatoria and other institutions for attacking the problem of the tuberculous, and to spend also a million a year for running these institutions and treating sufferers in their homes. But it may be asked what else has been done towards materialising these pious proposals? The recommendations of the Astor Departmental Committee on Tuberculosis have to all intents and purposes been simply ignored. This committee had the assistance of several well-known medical men, and presented a report which contained the outlines of a scheme for dealing in a comprehensive manner with consumptives all over the country. Grants were to be distributed by the Local Government Board with the consent of the Treasury, who were to consult the Insurance Commissioners before giving their consent. Plans for dealing with the whole country were to be drawn up by the councils of boroughs and counties with a view to the establishing of dispensaries which should eventually be incorporated in a complete and far-reaching scheme. But, again, it must be asked, has anything at all been done? If the consumption crusade which the Government is to initiate is under way, the public should be told what steps have been taken, for the sooner public attention is concentrated on this matter the better.

### SATURDAY FUND OR SOCIETY INSURANCE?

The secretaries of the Hospital Saturday Fund have issued an official explanatory pamphlet on its relations to and its position in regard to the National Insurance Act. This sets forth in parallel columns the benefits which the Act gives and those which the Fund provides. No provision is made in the Act for medical attendance and medicines required by the dependants of an insured person. The subscribers to the Saturday Fund obtain the benefits of treatment, for themselves and their dependants, at the institutions to which the Fund makes annual grants. The wives and families of the workers will, therefore, still have to rely on the work of this Fund and similar organisations to provide them with the means of treatment in times of illness. The pamphlet also shows that the Fund assists subscribers to obtain dental and convalescent home treatment, and that it does most excellent work in arranging for national instruction in first-aid, nursing, and hygiene—all matters which the Act does not deal with. The Act does not apply to persons under sixteen or over seventy years of age; the Fund excepts nobody. In conclusion, the secretaries of the Fund particularly emphasise a few points on which it is well to lay stress. These points are the following: No compulsory provision has been made in the State scheme for payments to the hospitals. As practically no provision is made for dependants—young people under sixteen and old people over seventy—all existing agencies, such as the Hospital Saturday Fund, are required as urgently as ever, and must continue their work. Insured persons also receive no benefits during certain probationary periods. Now that medical attendance is to be provided for workers, a larger number of cases than ever before are likely to require the special surgical treatment which only hospitals can provide; therefore more money must be available if the hospitals are to cope with the work. Many members of trade unions and friendly societies are now generous contributors to the Fund. As under the Act they will in many cases receive greater benefits without any additional contributions it is expected that they will not only continue their support, but bring the merits and claims of the Fund to the notice of their fellow-members. Much of the work carried on by the Fund is untouched by the Act. The pamphlet states that the Act will not diminish the necessity for the further activity of the Fund. Rather, if one may take the present chaos that prevails in the establishment of the machinery of insurance as a guide, it will increase the need for outside efforts regularly organised and economically controlled. The Hospital Saturday Fund has done so much good that it is unthinkable that it will lose subscribers owing to a belief that it is competing against the Act. Such a belief is absolutely groundless, and hospital workers will do well to impress upon their supporters how necessary it is to assist the Fund in every possible way. The little explanatory pamphlet will make the position quite clear.

### A COTTAGE HOSPITAL NOT TO BE ENVIED.

Mrs. Louisa Darell-Brown, of Hastings, and of Victoria Street, S.W., has left, subject to her husband's life interest, £12,000 to the St. Catherine's Clun Valley Cottage Hospital, established by her at Clun, Salop. Cottage hospitals are not often so fortunate as this institution at Clun. It was founded in 1893, and indeed has more than one point of interest. Mrs. Darell-Brown has been its parent from the first, and this large bequest of hers is only the testamentary recognition of her intention to provide as amply for its future after her death as she had done before. Designed by Mr. F. R. Kempson to contain six beds, and managed, under a trust deed, by a committee, the institution relied virtually on Mrs. Darell-Brown's support alone. That such individual support of any foundation with a public object is open to criticism no one with experience of administration or of charitable foundations generally can possibly deny. A privately maintained cottage hospital has on the face of it many advantages. It exaggerates the blessings of charity into a pauperisation of the neighbourhood. Its independence of local support may easily breed that indifference which is the soil in which bad management best thrives. It buys immunity from criticism by having little workaday claim on the goodwill and co-operation of the locality. Probably, as has been a feature of this institution, it issues no report. Everything, in short, is in a conspiracy to make efficiency as difficult as possible. Under these circumstances few cottage hospital managers will envy the Clun Valley institution the latest benefaction which the caprice of a rich woman, however well-intentioned, has thrown in its way.

### HOSPITAL ALMONERS AND THE INSURANCE ACT.

The Insurance Commissioners invite applications from women for the posts of inspectors (at a salary of £300, rising to £400), assistant inspectors (£100, rising to £300), and Health Insurance officers (£80, rising to £150). The appointments are permanent, and the women selected will have to give their whole time to the work. Candidates must be over twenty-five and under fifty years of age, and the final selection will be made on the results of a written examination to be conducted by the Civil Service Commissioners. The qualifications required are general administrative experience, especially in connection with public health work, special knowledge of industrial conditions, experience in dealing with bodies of employers or employed persons or in friendly society, trade union, or insurance work. This should give scope to hospital almoners whose work is essentially on the same lines as those mentioned by the Commissioners' memorandum and to school and district nurses who have had experience of visiting work generally. It would be well if the Commissioners stated what would be the status of these minor officials. Are they Civil servants and as such entitled to pension, or are they merely extra departmental officials?

### A STARTLING INNOVATION.

The Council of the British Medical Association recommends that practitioners in engaging locumtenents or assistants should insist that, previous to engagement, they shall have signed both the Undertaking and the Pledge of the Association; also that members of hospital staffs having influence in the selection of practitioners to fill resident appointments should do what they can to secure that those appointed shall have signed both these documents. The Association have been so completely terrified by the Insurance Act that it may be pardoned for losing its head, figuratively speaking. But medical practitioners who are in favour of the principles of toleration which have hitherto guided the profession in this country will regret to see it embark upon methods of coercion such as are foreshadowed in this recommendation. In any case, they may rest assured that the committees of hospitals will look to it that no undue influence shall be brought to bear upon candidates for hospital appointments to make them subservient to the Association. It is refreshing to see that the official organ of one of the largest medical schools in England, whose editor has had the moral courage to point out the risk of the recent policy of trying to force syndicalist methods upon the profession, vigorously protests against the recommendation. It is still more gratifying to find that few medical men have demanded the oath of allegiance from locumtenents, and that in no case has the refusal on the part of the locum to give such a pledge led to the breaking off of negotiations.

### "DUMPING BODIES" IN HONG KONG.

The people of Hong Kong seem likely to have the plague, like the poor, always with them. Ever since 1894, the year in which it first broke out in serious epidemic form, this troublesome disease has appeared in more or less grave proportions. In 1894 the deaths at one time reached over one hundred per day, and although (thanks to the tremendous efforts to remove sources of infection by sanitary measures) it has never since attained such a disastrous figure, yet science has failed to stamp out the disease or even materially to reduce the mortality from it. This summer it is again rampant, 199 cases being reported in the week; and the Chinese are resorting to their custom of dumping the bodies in order to escape the cost of burial and disinfection of the houses. In a single day (May 26) no fewer than eleven corpses were picked up in different parts of the Colony. This practice, of course, is one of the main causes of the spread of the disease, but the Colonial authorities seem powerless to check it. The neighbouring Portuguese Colony of Macao is also suffering from plague this year, though it is usually freer from it than Hong Kong. A plague hospital has been opened in Macao, and the Canossian Sisters of Mercy are giving their services free. This act is really a Christian one, for these Sisters have been most hardly treated by the Republican authorities, who ruthlessly ejected them from their institution.

### THE QUALIFICATIONS OF A HOSPITAL TREASURER.

The recent election of Mr. Woolmer White to the post of Treasurer to the Royal Portsmouth Hospital provoked a good deal of discussion as to the qualities which it is desirable for a candidate for such an office to possess. It was urged, for instance, that a gentleman with a working knowledge of banking should be appointed, and that, in fact, the hospital's bankers might continue, as had been the case with Mr. White's predecessor, to offer a member of their staff for the post. On the other hand, it was argued that a gentleman likely to be a permanency on the committee should be appointed. The Mayor, Alderman Sir Scott Foster, was emphatic that the hospital should retain its right to nominate the bank, since freedom of banking was an advantage in getting better terms. A good deal of discussion was aroused, some members fearing that Mr. White would not be able to attend the committee meetings with unfailing regularity. He was, however, elected by a majority of three over Mr. Knowles, the bank manager. The Mayor has also proposed a round-table conference between the governing body and the local authorities. Evidently the hospital is beginning to wake up to the gravity of its position.

### MENTAL PATIENTS IN WORKHOUSE WARDS.

One of the Commissioners in Lunacy, Mr. Trevor, has reported to the Chapel-en-le-Frith Guardians the presence of seventeen mentally afflicted persons in the workhouse and the infirmary. The gravaman of his report lies in the statement that he found the majority of these patients "all," we are told, "of the troublesome class," in the general wards of the workhouse. Among those not permanently detained he observed "a pronounced melancholic" and "a morose-looking person," who, in a fit of temper, had attempted to strangle an aged inmate. The Commissioner's comment was that it is unfair to expect the small staff of a workhouse to be responsible for patients who should at once be sent to a mental hospital. The report is now in the hands of the Medical Officer, and we hope that the public in Sheffield will back him up in insisting upon the Chapel-en-le-Frith Guardians at once showing cause why Mr. Trevor's report should not be carried out without delay.

### THE VALUE OF PRINTED FORMS.

Elsewhere in this issue appears an interesting article dealing with the question of "Economy of Labour in Hospital Management." The writer, who has taken pains to marshal his facts in such a way that they cannot fail to make an impression on the reader, gives full details of the forms used by his institution, and the paper raises the collateral question how far stereotyped printed cards are really economical in saving time and trouble in the working of a large hospital. Doubtless there are many workers who have views on the subject which may be of general interest to their institutional colleagues. We remember that the subject was debated some years ago at an American hospital conference, and that the opinion of the majority there was that such printed forms were absolutely indispensable, and greatly facilitated the work of the secretary's clerks. Nevertheless, there are certain objections to their use, the chief of which is probably the fact that where they are always used the secretary's office loses its individual character, and becomes a mere registering bureau. What have our readers to say on the subject?

### DEATH OF A WELL-KNOWN MEDICAL AUTHOR.

The death on June 28 of Dr. William Murrell removes from the profession one whose written word had made his name familiar to several generations of students. Dr. Murrell had many titles to medical distinction, but it is as the author of "What to do in cases of poisoning" that his name was so widely known. He wrote many other books upon professional subjects, but the tiny pocket manual referred to was easily the most successful of them, and passed through edition after edition. The humours with which it was besprinkled seemed to appeal very strongly to those for whom this book was written, though they were not in the least its real title to popularity. Dr. Murrell was at the time of his death senior physician to Westminster Hospital, in which capacity he succeeded the late Sir William Allchin. He was one of the few hospital physicians in London, perhaps the only one left at a teaching hospital, whose medical degree was that conferred by the University of Brussels. Educated at University College Hospital, he became L.S.A. in 1874, M.R.C.S. in 1875, and F.R.C.P. in 1883. He had also examined in Materia Medica at the Universities of Glasgow and Edinburgh, and at the Royal College of Physicians, London.

### A COMMITTEEMAN AND ANTIQUARY.

Bristol has lost a friend of her public medical institutions in the person of the late Mr. Robert Hall Warren, who died recently in his eightieth year. For many years he served actively on the committee of the Bristol Royal Infirmary. Conscientious and of a practical turn he served his city in more than one capacity, and held various honorary positions as secretary and treasurer of artistic and antiquarian associations. His father-in-law was Mr. Isaac Leonard, a well-known Bristol surgeon of the Early Victorian era, and his brother-in-law, the late Mr. Crosby Leonard, was a popular Bristol practitioner up to the eighties.

### CO-ORDINATION OF LONDON'S DISPENSARIES.

The Metropolitan Borough Councils are endeavouring to arrange for a conference to discuss the whole question of the Parliamentary grants for sanatoria and tuberculosis dispensaries. By this means it is hoped to secure uniformity of action throughout London. The Lambeth and Holborn Borough Councils are in favour of the conference. At Hampstead the Council is strongly of opinion that in the Metropolis the provision of the dispensaries should be undertaken by the Borough Councils either singly or in groups. At the same time the London County Council is drafting a general scheme for the county area. It is hoped that in the multitude of opinions a uniform system may be adopted which will remove any dangers of overlapping.

### THE BRITISH HOSPITALS ASSOCIATION.

At a meeting of the Council of this Association at St. George's Hospital on the 4th instant the following resolution was proposed by Mr. Tait, seconded by Mr. W. G. Carnt, and unanimously carried: "That the Council having considered the available information as to the treatment in voluntary hospitals of insured persons suffering from tuberculosis resolved to adjourn the discussion until July 15 in the hope that further information from the Local Government Board and the Insurance Commissioners may then be available." A discussion then took place with regard to placing the nursing staff on the reduced scale, and it was moved by Mr. W. J. Morton, seconded by Mr. Thies, and carried unanimously: "That in the opinion of this Council the hospitals should not make any application for a special order under Section 47, but in the meantime should place their nursing and domestic staff under full scale of payment." A further resolution, moved by Mr. Thomas Hayes and seconded by Mr. Stewart Johnson, was also carried unanimously, viz.: "In the opinion of this Council it is undesirable that the employer should pay the employee's contribution." The Council especially draw the attention of hospital managers generally to Clause 15, Sub-section 3, which is important in regard to matters which formed the basis of discussion at the meeting on the 4th instant. We are glad to find that the opinion of hospital managers generally, both in England and Scotland, is practically unanimous in regard to the points covered by the resolutions.

### FIFTY YEARS' SAVINGS FOR MIDDLESEX HOSPITAL.

Another of those odd yet relatively well-known instances of personal gifts by unexpected donors occurred at the Middlesex Hospital on June 2. An old lady, with a bag containing £100, called at the hospital, and explained to Mr. Clare Melhado, the secretary-superintendent, that she had come in memory of an earlier visit over fifty years before. During the half-century which has elapsed her gratitude has been equalled by her means to express it, and her present is remarkable for its generosity and live conviction. Such gifts best express the patient's relation to the spirit which the voluntary system has so widely inspired. A hospital secretary of our acquaintance, now retired, is fond of telling how an old lady entered his office, refusing to be hurried in explaining what was the object of her visit. Diagnosing a present benefactor or a future testator, the secretary, as his genial and well-tried custom was, offered the old lady some light refreshment, and begged her to make herself comfortable in his arm-chair. Taking him at his word, and being somewhat overcome by the heat, the old lady asked if she might take off her boots. He begged her to do so, and after she had partaken of refreshment took her leave with many expressions of thanks for his courtesy. He was able to trace her, and his good manners and patience were rewarded when, some years later, she left to his hospital a large sum.

### SHEFFIELD CRIPPLES' HOME.

A competition limited to architects practising in Sheffield, to ensure a good design for the projected Home for Cripples in the New Rivlin Valley Road, was announced in our issue for March 30. The assessors, Mr. E. M. Gibbs, F.R.I.B.A., in conjunction with the City Architect, Mr. F. E. P. Edwards, have now made an award in favour of a design submitted by Mr. Arthur W. Kenyon, A.R.I.B.A.

### THE LONDON COUNTY COUNCIL MEDICAL STAFF.

Pursuant to the recommendations of the Education Committee, the London County Council is now calling for medical men and women to fill twenty-eight newly created appointments in its Public Health Department. Nine of the appointments will be on the permanent staff, two at a commencing salary of £500 a year rising to £600, and seven at a commencing salary of £400 rising to £500. The remaining nineteen appointments will be for one year at a salary of £400 a year, "and the continuance of such temporary appointments after that term will depend upon the decision at which the Council may arrive with regard thereto." The work of the new medical officials will lie chiefly in the schools, and it is definitely stated that candidates who have had special aural or ophthalmic experience, who hold diploma in Public Health and have already had experience in the medical examination of school children, will have their applications specially considered. The last day for sending in applications is Monday next, July 15. The new appointments should prove acceptable to many young practitioners who do not possess the necessary capital to embark on private or consulting practice, for the school work that the newly appointed doctors will be called upon to do is varied and interesting.

### THE PRESENT-DAY RATING OF HOSPITALS.

"Is it not time," asks Mr. Robert J. Bland, secretary of the Royal London Ophthalmic Hospital, "for the Local Government Board to arrange that hospitals should be uniformly assessed, or that they should be exempt from rates?" And he backs his question by remarking that "for nearly thirteen years the Royal London Ophthalmic Hospital has been burdened first by St. Luke's parish authorities and of later years by the borough of Finsbury by an assessment out of all proportion to that of any other hospital in London." Mr. Bland further states that "during the twelve years ended December 31, 1911, the Royal London Ophthalmic Hospital has paid no less a sum than £10,467 in rates, and that out of some 80 voluntary hospitals in London only six hospitals paid more in rates during that period," and he records that an attempt made in 1910 to awaken public sympathy by an appeal, which had proved successful ten years previously when his hospital actually was summoned for failing to pay, failed to repeat itself. This, of course, is only a glaring example of an old grievance, and we are afraid that Mr. Bland's despairing appeal to the Local Government Board will produce no result. For, while urging individual hospital secretaries to secure all the rebate which their individual enterprise can effect, the blunt truth is that justice will never be done in the matter of rating, either to institutions or to individuals, until the whole system of rating is radically revised. But the more individual agitation and protest there is on the part of such sufferers as hospitals the sooner the chance for a general measure of reform.

### THE NEW BANGKOK UNIVERSITY.

Fired probably by the example of Hong Kong, the Siamese Government announces the early establishment of a University at Bangkok. It is to be on modern lines, and will have eight faculties, of which medicine is placed in the forefront. It is greatly desired that medical science shall be promoted in order to provide for the needs of the country, which up to the present have been met by the Medical College at Bangkok and the Siriraja, Burabba, Bangrak, Sam Sen, and Debsirindi Hospitals. The scheme for the University has been sanctioned in its general outlines by the King of Siam.

### SECRET REMEDIES.

Further testimony in support of the case against secret remedies was given by Mr. E. F. Harrison, B.Sc., F.I.C., at the last sitting of the Select Committee, and Mr. Harrison's evidence, taken as a whole, forms a very powerful indictment against quackery of this particular kind. One of the members of the Committee was desirous of ascertaining whether it would be possible for an absolutely worthless medicine to live for any length of time. "For instance," he asked, "do you think that a mixture of sugar and water could be launched as a paying proprietary medicine?" Mr. Harrison's answer was, "I can give you a case of a medicine which consisted of the water without the sugar, which had an extensive sale." The reference was, of course, to Count Mattei's remedies, which were recommended for all manner of diseases. It is well known that some of the manufacturers, whose products have been analysed and the results published by Mr. Harrison, state that the analyses are incorrect; asked whether he would accept the statement of a manufacturer to the effect that his preparation contained something which the analyst had not found, Mr. Harrison replied that he would accept the statement, but would not attach much importance to it unless he could cross-examine the manufacturer. As a matter of fact, it is highly improbable that an analyst accustomed to the examination of pharmaceutical products would find it difficult to disentangle the ingredients of the majority of proprietary medicines; occasionally, of course, important ingredients might be overlooked, and occasionally manufacturers may add something to their mixtures which are merely designed to "fog" the analyst. On the whole, the best way to defeat quackery is, perhaps, to lay bare the secrets of the quacks. The evidence of Dr. Mary Sturge, the witness who followed Mr. Harrison, dealt solely with medicated wines, of which she has made a special study. Investigations have shown that some of the advertised "medicated" wines contain as much as 20 per cent. of alcohol, while many people who are induced by advertisements to drink them are under the impression that the wines are non-alcoholic.

### THIS WEEK'S DRUG MARKET.

Quiet conditions still prevail in the Drug market, and the holiday feeling seems to have set in already. The labour troubles at the docks, which are far from settled, also continue to place a check on business. Of price changes few have occurred during the week. In consequence of an advance in the price of quicksilver, makers of corrosive sublimate, calomel, and other salts of mercury have advanced their prices by 1½d. per pound. Eucalyptus oil is rather dearer owing to a shortage of supply in Australia. The position of the Opium market is unchanged, and buyers are still holding off. Codeine is unaltered since last week's reduction in price, and morphine is without change, but with a slight tendency downwards. Rhubarb is still inclined to advance in price.

### TO OUR READERS.

Contributions are specially invited from any of our readers to these columns. They should deal with topical subjects and news. They must be authenticated for the information of the Editor only. The minimum payment if published is 5s. There is no hard-and-fast rule as to space, but notes of about twenty lines in length are preferred.

